# Supplementary material for: Resolving the full spectrum of human genome variation using Linked-Reads
Source: Genome Res. 2019 Apr;29(4):635–45. doi: 10.1101/gr.234443.118 (PMC6442396; doi:10.1101/gr.234443.118)
Supplement: Supplemental Material [file supp_gr.234443.118_Supplemental_longranger-2.2.zip › longranger-2.2.tar.bz2!/lr2.2-public/lib/stan/cmdstan/stan/doc/api/html/check__vector_8hpp_source.html]

Stan: src/stan/math/error\_handling/matrix/check\_vector.hpp Source File


|  |  |
| --- | --- |
|  | Stan  2.5.0  probability, sampling & optimization |


- Main Page
- Related Pages
- Namespaces
- Classes
- Files

- File List
- File Members

All Classes Namespaces Files Functions Variables Typedefs Enumerations Enumerator Friends Macros Pages

- src
- stan
- math
- error\_handling
- matrix

check\_vector.hpp

Go to the documentation of this file.

1 #ifndef STAN\_\_MATH\_\_ERROR\_HANDLING\_\_MATRIX\_\_CHECK\_VECTOR\_HPP

2 #define STAN\_\_MATH\_\_ERROR\_HANDLING\_\_MATRIX\_\_CHECK\_VECTOR\_HPP

3

4 #include <sstream>

5 #include <stan/meta/traits.hpp>

6 #include <stan/math/error\_handling/dom\_err.hpp>

7 #include <stan/math/matrix/Eigen.hpp>

8

9 namespace stan {

10  namespace math {

11

12  // NOTE: this will not throw if x contains nan values.

13  template <typename T, int R, int C, typename T\_result>

14  inline bool check\_vector(const char\* function,

15  const Eigen::Matrix<T,R,C>& x,

16  const char\* name,

17  T\_result\* result) {

18  if (x.rows() == 1 || x.cols() == 1)

19  return true;

20

21  std::ostringstream msg;

22  msg << name << " (%1%) has " << x.rows() << " rows and "

23  << x.cols() << " columns but it should be a vector so it should either have 1 row or 1 column";

24  std::string tmp(msg.str());

25  return dom\_err(function,

26  typename scalar\_type<T>::type(),

27  name,

28  tmp.c\_str(),"",

29  result);

30  }

31

32  }

33 }

34 #endif

dom\_err.hpp

stan::math::dom\_err

bool dom\_err(const char \*function, const T &y, const char \*name, const char \*error\_msg, const T\_msg error\_msg2, T\_result \*result)

**Definition:** dom\_err.hpp:36

stan::math::check\_vector

bool check\_vector(const char \*function, const Eigen::Matrix< T, R, C > &x, const char \*name, T\_result \*result)

**Definition:** check\_vector.hpp:14

stan::scalar\_type::type

scalar\_type\_helper< is\_vector< T >::value, T >::type type

**Definition:** traits.hpp:139

traits.hpp

Eigen.hpp


---

[ Stan Home Page ]

*© 2011–2014,
Stan Development Team.*
